# Identification and molecular characterization of simian endogenous retrovirus in *Macaca fascicularis* and *Macaca nemestrina* from captive breeding facilities in Bogor, Indonesia

**DOI:** 10.14202/vetworld.2022.1827-1834

**Published:** 2022-07-27

**Authors:** Fitri Luthfianti Nur Annisaa, Uus Saepuloh, Diah Iskandriati, Joko Pamungkas

**Affiliations:** 1 Primatology Graduate Study Program, Graduate School of IPB University, Bogor 16128, Indonesia; 2 Primate Research Center, IPB University, Bogor 16128, Indonesia; 3 Department of Animal Infectious Diseases and Veterinary Public Health, Faculty of Veterinary Medicine, IPB University, Bogor 16680, Indonesia

**Keywords:** *Macaca fascicularis*, *Macaca nemestrina*, retrovirus, simian endogenous retroviruses

## Abstract

**Background and Aim:**

Endogenous retroviruses (ERVs) found in all vertebrates, including non-human primates (NHPs), are known to be genetically inherited. Thus, recent studies have explored ERVs for human immunodeficiency virus vaccine development using human ERV (HERV) due to the hypervariability of exogenous retroviruses which cause conventional vaccines to be ineffective. HERV was also found to be able to induce an immune response in cancer patients. This study aimed to identify and molecularly characterize ERVs from Indonesian NHPs: *Macaca fascicularis* and *Macaca nemestrina*. Then, we described the phylogenetic relationship of these isolates with those of the simian ERVs (SERVs) characterized in other species and countries.

**Materials and Methods:**

First, 5 mL of whole blood samples was taken from 131 long-tailed macaques and 58 pig-tailed macaques in captive breeding facilities at Bogor, Indonesia, for DNA extraction. Next, the DNA samples were screened using the SYBR Green real-time polymerase chain reaction (PCR) technique with specific primers for *env* (simian retroviruses [SRV]1-5 7585U19 and SRV1-5 7695L21). Positive SERV results were those with cycle threshold (CT) values < 24 (CT < 24) and melting temperature (TM) ranges of 80°C–82°C. Then, whole-genome nucleotide sequences from two pig-tailed macaques samples detected as positive SERV were generated using a nucleic acid sequencing technique which utilized the walking primer method. Subsequently, the sequences were analyzed using bioinformatics programs, such as 4Peaks, Clustal Omega, and BLAST (NCBI). Subsequently, a phylogenetic tree was constructed using the neighbor-joining method in MEGA X.

**Results:**

SYBR Green real-time PCR amplification results indicated that SERV (Mn B1 and Mn B140910)-positive samples had CT values of 22.37–22.54 and TM of 82°C. Moreover, whole-genome sequences resulted in 7991 nucleotide sequences, comprising long terminal repeat, *gag*, *pro*, *pol*, and *env* genes identical between the sequenced samples. Furthermore, the phylogenetic tree results indicated that both samples from *M. nemestrina* had 99%–100% nucleotide identities to the Mn 92227 sample identified at the National Primate Center University of Washington (NaPRC UW) which was imported from Indonesia in 1998, confirmed as a novel SERV strain. The phylogenetic tree results also indicated that although SERV whole-genome nucleotide and *env* amino acid sequences were clustered with SRV-2 (identity values of 82% and 79%, respectively), they had a 99%–100% nucleotide identity to Mn 92227. Meanwhile, the *gag*, *pro*, and *pol* amino acids were clustered with SRV-1, SRV-3, SRV-4, SRV-5, SRV-8, and SERV/1997, with 82% and 88% identity values.

**Conclusion:**

Based on the SYBR Green real-time PCR profiles generated, similarities with Mn 92227 were observed. Subsequent phylogenetic analysis confirmed that both samples (Mn B1 and Mn B140919) from pig-tailed macaques in the country of origin were novel SERV strains at NaPRC UW. Therefore, it could be used in biomedical research on ERVs.

## Introduction

Endogenous retroviruses (ERVs) are found in all vertebrates, including non-human primates (NHPs) [1–3]. Although their sequences are genetically inherited and have high copy numbers in the genomes of all species, most strains are unable to produce viruses [4, 5]. Therefore, ERVs are assumed to be inherited from parents through oocytes or embryos infected with exogenous retroviruses. Subsequently, nonpathogenic microorganisms are considered to undergo coevolution between the host and the pathogen, resulting in various ERVs [4].

While exogenous retroviruses are horizontally transmitted, ERVs are transmitted vertically from parents to offspring. For example, type D retroviruses in exogenous and endogenous forms were first discovered in Old World monkeys (OWMs). Exogenous simian retrovirus type D infection in Asian non-human primate (NHP) cause immunodeficiency with many characteristics of acquired immune deficiency syndrome (AIDS) in human, or commonly in NHP referred to as simian acquired immune deficiency syndrome (SAIDS). It has been reported that ERV defects caused by some deletions or mutations could cause it to lose the ability to become infectious viruses, to be non-pathogenic, to be unable to transform cell cultures, and to not contain oncogenic genes. Complete genomes of endogenous proviruses from OWM include baboon endogenous virus [6], simian ERV (SERV) [7], Papio cynocephalus endogenous virus isolated from yellow baboons [8], and rhesus ERV-K/SERV-K1 [9].

Previously, Grant *et al*. [10] detected novel SERV strains in five *Macaca nemestrina* samples imported from Indonesia. Serological test results indicated that antibody responses cross-reacted with experimental samples (SRV-5 and SRV-2). Moreover, although the experimental samples had melt temperature (TM) values at 80°C–81.5°C and cycle threshold (CT) values at <24, peripheral blood mononuclear cells and plasma showed no infectious virus results when cultured with Raji cells for more than 4 weeks. Subsequently, no research on novel SERV strains passed from parent to offspring has been conducted.

NHP genus *Macaca*, such as *Macaca fascicularis* and *M. nemestrina*, is used as animal models in biomedical research due to their relevance to humans [11] and physiological, anatomical, and immunological similarities to humans. Therefore, their uses have been relevant for studying complex human diseases. For example, Holloway *et al*. [12] reported that gorillas could serve as a model for understanding human retrovirus evolution and pathogenesis.

Human immunodeficiency virus (HIV) type 1 is considered to have evolved from the simian immunodeficiency virus (SIV), SIVcpz. Experimental infection of Asian primates with SIV showed that the resultant clinical signs were similar to those in HIV-infected humans. Therefore, primates can be used as animal models for understanding the origin and pathogenesis of HIV, *in vivo* testing of new vaccines, and developing prevention/treatment strategies for HIV [13]. Recent studies of human ERV (HERV) also proposed another approach to study HIV vaccines based on the immune-specific responses of anti-HERV, which cross-reacted with T-cells due to HIV infection [14, 15] and investigate antitumor or anticancer therapies [16, 17].

This study aimed to identify and characterize novel SERV strains in *M. fascicularis* and *M. nemestrina* from a captive breeding facility in Bogor, Indonesia, at a molecular level. The results obtained should provide updated information on the presence of SERV in Indonesia, including their molecular characterization data and genetic relationships with SERV strains in other countries. Another possible use of our results is that the data can be used in biomedical research on ERVs.

## Materials and Methods

### Ethical approval

This study used whole blood samples of long-tailed macaques (*M. fascicularis*) and pig-tailed macaques (*M. nemestrina*). The Institutional Animal Care and Use Committee approved the use of these animals and procedures for blood collection (PSSP-LPPM IPB University number IPB PRC-18-B008).

### Study period and location

The study was conducted from February to November 2020 in the Biotechnology Laboratory, Primate Research Center of IPB University, Bogor, Indonesia. The samples were collected from PT Wanara Satwa Loka in Cikarawang, Bogor, Indonesia, and the PSSP-LPPM IPB University Breeding Facility in Dramaga, Bogor, Indonesia.

### Animals

In this study, whole blood (~ 5 mL) from 131 long-tailed macaques (15 males and 116 females) and 58 pig-tailed macaques (seven males and 51 females), both from PT Wanara Satwa Loka in Cikarawang, Bogor, Indonesia, and the PSSP-LPPM IPB University Breeding Facility in Dramaga, Bogor, Indonesia, was used. Before sample collection, all animals were anesthetized with ketamine (10 mg/kg body weight). Then, approximately 5 mL of blood sample was collected from the femoral vein of each animal and put into a blood tube containing ethylenediaminetetraacetic acid during general examinations.

### Nucleic acid isolation

According to the manufacturer’s protocol, DNA from blood samples was extracted using a QiaAmp™ DNA Extraction Blood Mini Kit (Qiagen, Hilden, Germany). Then, the DNA concentrations were measured using NanoDrop™ One (Thermo Scientific, Massachusetts, US).

### DNA amplification

Real-time polymerase chain reaction (PCR) amplification was performed using primers for *env* genes designed by the National Primate Research Center, University of Washington, USA (Richard Grant, personal communication by Uus Saepuloh): SRV1-5 7585U19 (5′-CTGGWCAGCCAATGACGGG-3′) and SRV1-5 7695L21 (5′-CGCCTGTCTTAGGTTGGAGTG-3′). The PCR reaction comprised 0.5 mL each of the 10 pmol/μL forward and reverse primers, 10 μL Ssofast™ EvaGreen^®^ Supermix, 2.5 μL DNA template, and 6.5 μL nuclease-free water. Then, amplification was conducted using a real-time PCR machine Bio-Rad iQ™5 (Bio-Rad, California, US) under the following conditions: Pre-denaturation at 98°C for 2 min, 40 cycles of denaturation at 98°C for 10 s, and annealing/elongation at 64°C for 20 s. Next, melting curve analysis was conducted at 60°C and 95°C, increasing every 0.5°C. SERV suspected samples were those with melting TM ranges of 80°C–82°C and CT values <24 [10].

Subsequently, whole-genome characterization of SERV was performed for suspected positive samples using the walking primer method developed at the Biotechnology Laboratory PSSP-LPPM IPB University (Table-1). The PCR reaction comprised 1 μL each of 10 pmol/μL forward and reverse primers, 12.5 μL GoTaq^®^ Green Master Mix (Promega, Wisconsin, US), 2.5 μL DNA template, and 8 μL nuclease-free water. Then, amplification was conducted using a conventional PCR machine GeneAmp^®^ PCR System 9700 (Applied Biosystems, Massachusetts, US) under the following conditions: Pre-denaturation at 94°C for 3 min, 40 cycles of denaturation at 94°C for 30 s, annealing (according to the TM of each primer) for 30 s, elongation at 72°C for 2 min, and extension at 72°C for 7 min. Finally, after the PCR products had been electrophoresed in 1% agarose gel and visualized using SYBR™ Safe (Invitrogen, Massachusetts, US) under UV Gel Doc 2000 (Bio-Rad), the bands were analyzed using the Quantity One^®^ program (Bio-Rad), followed by amplicon sequencing at 1^st^ BASE Laboratories Sdn. Bhd. (Malaysia).

**Table 1:** List of primers used for whole-genome sequencing in this study.

Primers	Primer sequence	Ta	Amplicon (bp)	Reference
SERV1F	5’TGTCCGGAGCCGTGCAGCCCG3’	56	625	Biotechnology Lab. PSSP-LPPM IPB design
SERV880R	5’GGTCCATTTCTCTATCTGGTG3’	56	700	
SERV441F	5’GCCGACAGTTAAAGTGAAAG3’	56	700	
SERV1046F	5’AGGATGAGGCAGCAAATATC3’	56	700	
SERV1670F	5’AAATGGCTGGGATTTTGATA3’	56	700	
SERV2369R	5’GAAACTGACTGCCCCATAAG3’	56	700	
SERV2243F	5’AAAACATTGGGCTAATGAGTG3’	56	700	
SERV2744F	5’AGTTTCGGGTCTTCAGACAT3’	56	700	
SERV3273F	5’TACGTGGAAGTCAGATGAGC3’	56	700	
SERV3972R	5’TCTACGGATAGTTGCTTTTTGA3’	56	700	
SERV3802F	5’GGGAAAAATGGACAACAAGT3’	56	700	
SERV4421F	5’TCCATTGGTTAATGCAAAAT3’	56	700	
SERV4984F	5’GATCAAGCACAAACTGCTCA3’	56	700	
SERV5346F	5’TGGTCCTGGATACACCTCTAA3’	56	700	
SERV6045R	5’ATGCAGTGTGAGAGGAACAG3’	56	700	
SERV5924F	5’GCGTGAAGCCCATTACAATAA3’	56	700	
SERV6245F	5’GGCCAGTAATGTCCCTACAG3’	56	700	
SERV6847F	5’TGCCCCAAATAGTTCAGTTT3’	56	700	
SERV7322F	5’CAAGATTAAACGCCTACAGGA3’	56	700	
SERV8021R	5’TTATATAGGCGGGCAGTAGG3’	56	700	

### Bioinformatics

First, nucleotide sequences were manually edited based on their chromatograms using the 4Peaks program (https://4peaks.en.softonic.com/mac). Subsequently, the alignment of nucleotide sequences was conducted using the Clustal Omega program, after which the SERV alignment results were analyzed using the NCBI website (https://blast.ncbi.nlm.nih.gov) by selecting BLAST-N to identify the closest relatives. Then, the nucleotide sequences obtained were further analyzed using the MEGA-X program (https://www.megasoftware.net/) and translated into amino acids through an ORF finder. Finally, genetic distance estimation was performed using the pairwise distance method with the *p*-distance model, followed by a phylogenetic tree construction based on the neighbor-joining (NJ) method with 1000 repetitions.

## Results

### SERV screening test by real-time PCR SYBR Green

**Figure-1:**
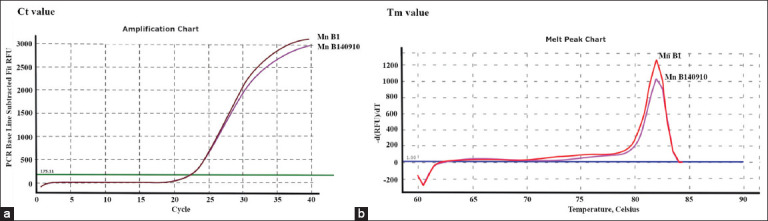
Characteristics of novel simian endogenous retroviruses based on real-time polymerase chain reaction of SYBR Green were shown: (a) Curve of cycle threshold value <24.0; (b) curve of temperature value at temperature range 80–82°C.

The detection of SERV-positive samples was based on proviral DNA amplification results, using *env* primer pairs (SRV1-5 7585 U19 and SRV1-5 7695 L21), which were TM characterized at 82°C and CT values of 22.37–22.54 (Table-2) and Figure-1). Subsequently, the positive results obtained for two samples from pig-tailed macaques were assigned identity numbers (Mn B1 and Mn B140910).

**Table 2:** Summary of SERV detection using SYBR Green real-time PCR technique on long-tailed macaque and pig-tailed macaque samples in breeding captivity in Bogor, Indonesia.

No.	Species	n	Sex	Positive	ID	CT value		TM value	
1	Long-tailed macaque	131	Male: 15 Female: 116	0 of 131	N/A	N/A	N/A	N/A	N/A
2	Pig-tailed macaque	58	Male: 7 Female: 51	2 of 58	Mn B1 Mn B1409190	22.54 22.41	22.52 22.37	82.0 82.0	82.0 82.0

SERV=Simian endogenous retroviruses, CT=Cycle threshold, TM=Melting temperature, PCR=Polymerase chain reaction

### Gene amplification

**Figure-2:**
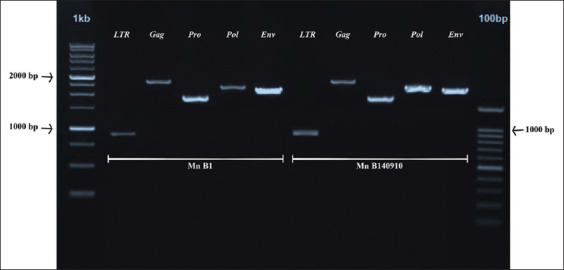
DNA visualization of simian endogenous retroviruses whole genome for target genes long terminal repeat (879 bp), *gag* (1928 bp), *pro* (1729 bp), *pol* (2243 bp), and *env* (2097 bp) in 1% agarose gel. 1 kb and 100 bp: DNA ladder.

PCR amplification of the target genes successfully yielded the following amplicons: long terminal repeat (LTR) (879 bp), *gag* (1928 bp), *pro* (1729 bp), *pol* (2243 bp), and *env* (2097 bp). The amplification results are presented in Figure-2.

### Gene mapping

**Figure-3:**
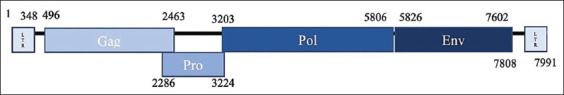
Genome mapping of simian endogenous retroviruses isolated from pig-tailed macaque of Mn B1 and Mn B140910. Nucleotide sequence position is indicated by number for each gene.

Subsequently, PCR amplification products (LTR, *gag*, *pro*, *pol*, and *env*) were sequenced to obtain the complete SERV genome sequence. MnB1 and Mn B140910 exhibited DNA lengths similar to the provirus of 7991 bp. Furthermore, the sequences comprised two LTRs (1–348 and 7808–7901 bp), *gag* (496–2463 bp), *pro* (2283–3226 bp), *pol* (3203–5806 bp), and *env* (5827–7602 bp) (Table-3). The genetic mapping results for Mn B1 and Mn140910 are presented in Figure-3.

**Table 3:** Summary of comparison of whole-genome nucleotide identities of the samples obtained in this study with those of SERV/1997 from baboon and other SRVs from GenBank (SRV-1-5 and SRV-8).

Target	Nucleotide position in proviral DNA	Nucleotide/amino acid length	Between samples* (%)	Identity		

				Mn 92227** (%)	SRV-2 (IFB9578) (%)	SERV/1997 and other SRVs in GenBank*** (%)
Whole genome	7991 nt		100	100	82	74–82
LTR-1	1–348	348	100	100	82	71–82
*Gag*	496–2463	1967/655	100	100	87	82–87
*Pro*	2283–3226	941/314	100	100	87	83–88
*Pol*	3203–5806	2603/867	100	99–100	87	82–88
*Env*	5827–7602	1776/591	100	100	79	65–79
LTR-2	7808–7901	93	100	100	81	75–81

* Mn B1 and Mn B140910,

** Mn 92227 NaPRC UW isolate that confirmed as novel SERV from pig-tailed macaque,

*** GeneBank access code M11841.1 for SRV-1 L47.1, KY235267.1 for SRV-2 isolate IFB9578, M12349.1 for SRV-3/ MPMV, NC_014474.1 for SRV-4 strain SRV/TEX/2009/V1, AB611707.1 for SRV-5 strain Y001, NC_031326.1 for SRV-8 strain SRV/SUZ/2012, U85505.1 for SERV/1997. MPMV=Mason-Pfizer monkey virus, SERV=Simian endogenous retroviruses, SRV=Simian retroviruses

### Phylogenetic tree

**Figure-4:**
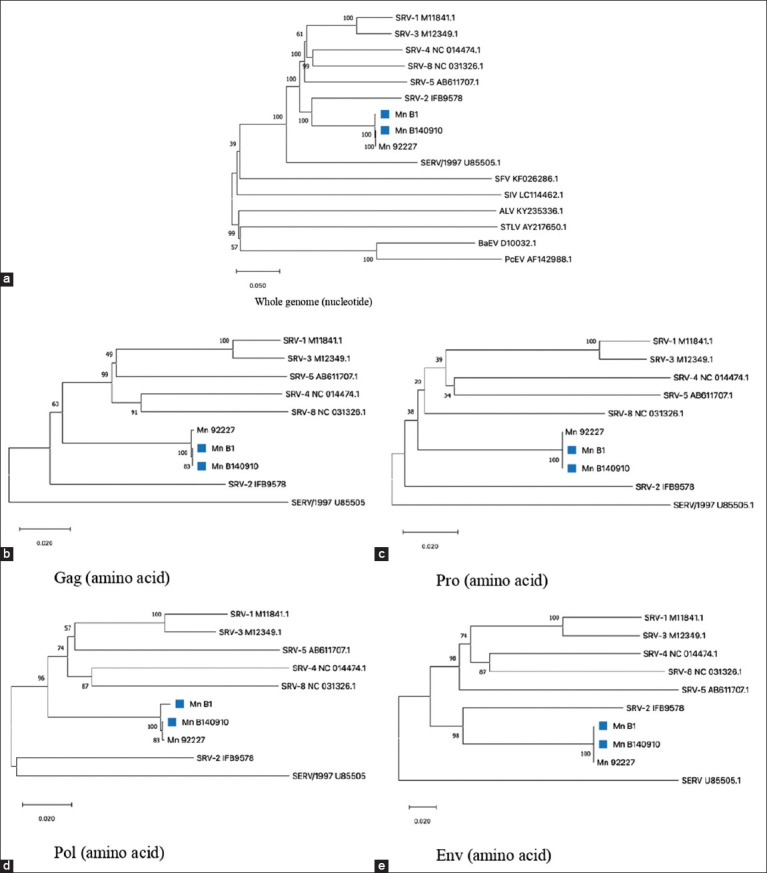
Phylogenetic tree construction of samples against other retroviruses for whole genomes (a); against baboon simian endogenous retroviruses (U85505.1), 92227, simian retroviruses (SRV) 1-5, and SRV-8 for *gag* amino acid (b), *pro* amino acid (c), *pol* amino acid (d), and *env* amino acid (e).

At a bootstrap value of 100%, phylogenetic analysis using the NJ method with 1000 bootstraps (Figure-4) revealed that the whole nucleotide genomes of MnB1 and MnB140910 were grouped with Mn 92227 (National Primate Center University of Washington [NaPRC UW]). However, *gag*, *pro*, and *pol* amino acid phylogenies separated the sequences from exogenous SRV-2 and clustered them with SRV-1, SRV-3, SRV-4, SRV-5, and SRV-8. Furthermore, amino acids of the *env* gene of MnB1 and MnB140910 grouped with exogenous SRV-2 had a bootstrap value of 98%, which separated and formed branches from other SRVs.

## Discussion

Suspected novel SERV genome sequences were obtained from two blood samples of *M. nemestrina*, after which they were assigned identity numbers: Mn B1 and Mn B140910. Subsequently, the targeted SERV genome regions were amplified at TM of 82°C and CT values of 22.37–22.54 based on a previous report by Grant *et al*. [10] for obtaining novel SERV SYBR Green real-time PCR profiles. Smaller CT values indicated a more significant number of amplified targets (amplicon) and *vice versa*. Grant *et al*. [10] also proposed that samples with CT values of approximately 23 should have 100,000 copies of amplicons per ng of DNA, similar to the PCR control DNA sample. Likewise, the animals experimentally infected with SRV-2 and SRV-5 showed CT values of 32–35, which were estimated to contain approximately 10–100 copies of amplicons per ng of DNA. Control DNA samples have highly conserved SERV genome regions with slower evolutionary rates and Ct values of 15-30 [18] According to Ma *et al*. [5], although ERVs have high copy numbers in their genomes that can be translated into RNA, most of them disintegrate and fail to produce infective viruses.

**Figure-5:**
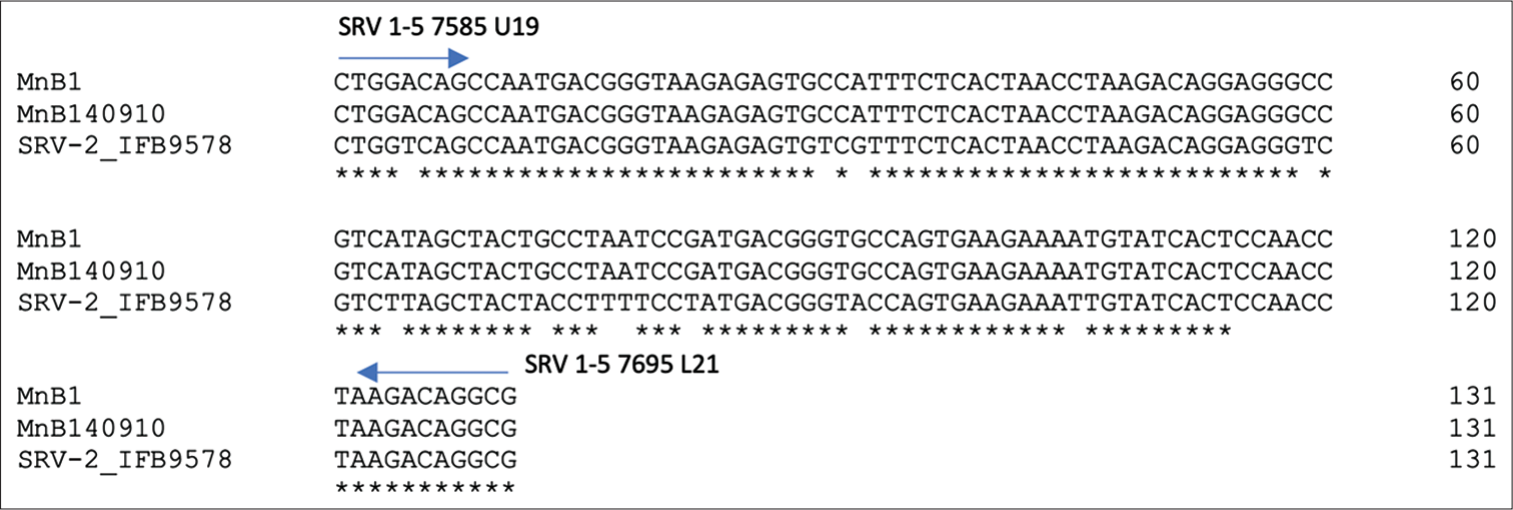
Alignment of novel simian endogenous retroviruses (Mn B1 and Mn B140910) and exogenous simian retroviruses (SRV) (SRV-2 IFB9578) at nucleotide positions 7585–7695 and variations (same nucleotides marked with*). The primary positions of SRV1-5 7585 U19 and SRV1-5 7695 L21 are marked with arrows.

A study also reported that the TM of SYBR Green real-time PCR amplification results for SRV serotypes could be grouped into two ranges: 79°C–79.5°C for exogenous SRV-2 and 80°C–82°C for endogenous SRV [9]. Similarly, at nucleotide position 7585–7695 (*env*), we observed 11 different nucleotide variables between novel SERV and exogenous SRV-2 that caused a shift in TM from 79°C–79.5°C to 80°C–82°C (Figure-5).

Suspected positive samples were amplified by PCR using the walking primer method designed at the Biotechnology Laboratory PSSP-LPPM IPB University. Primers were designed from novel Indonesian pig-tailed macaque SERV nucleotide sequences (Mn 92227), isolated at the NaPRC University of Washington (Richard Grant, personal communication by Uus Saepuloh). Based on SERV whole nucleotide sequences, we observed that Mn B1 and Mn B140910 samples were 99–100% similar. However, while these samples had the highest similarity to Mn 92227, confirmed as a novel SERV from pig-tailed macaque [10], they also had a similarity to SRV-2 of 82% (Table-3).

The LTR positions of samples were at 5′ (1–348) and 3′ (7808–7990) ends. While a “TGTCC” sequence determines LTR borders in other SRV-type D viruses, U5 (GGACA) was found at the other end of the LTR [7]. Furthermore, the presumed position of a “TATA” box was at nucleotide positions 209–215 (TATATAA). According to Zao *et al*. [19], the TATA or the Goldberg–Hogness box is a sequence region that functions as a transcriptional promoter. Moreover, the Poly-A signal (ATTAAA) was 17 bp downstream from the TATA box. Downstream (350–367) of the 5′LTR (TGGCGCCCAACGTGGGGC) is a conserved primary binding site, which allows cellular tRNA as a primer to bind and initiate negative-strand DNA synthesis during reverse transcription process. The positions 7791–7805 were located upstream of the 3′LTR polypurine tract (ppt) (AATAAAAAAGGGGGA), functioned as a primer for positive-strand viral DNA synthesis [7]. LTR has also been proposed to function in integrating viral DNA into the host chromosome, viral DNA synthesis, and viral gene expression [20]. Although the LTR of ERV contains many regulatory sequences, such as promoters, amplifiers, polyadenylation signals, and binding factor sites, it is often fragile as a transcriptional promoter compared with exogenous viruses [21]. In addition, it affects the expression of surrounding genes [22]. This study found a 19% (64/349) LTR nucleotide variation between exogenous and endogenous SRV strains.

*Gag* is translated at position 497, starting from codon ATG [7]. Furthermore, it has a proviral genome length of 1967 bp and encodes 655 amino acids. Amino acid sequence analysis revealed a 100% identity between the two samples and Mn 92227. Subsequent comparison with SERV from baboons also indicated that SRV1-5 and SRV-8 had an amino acid identity of 82%–87% (Table-3). In this study, one-stop codons were found in MnB1 and MnB140910. Therefore, we propose that the presence of a stop codon might cause differences in the structure of the *gag* sample compared with that of exogenous SRV.

Alternatively, *pro* has a length of 943 bp and encodes 314 amino acids. In a study, some overlapping sequences at downstream *gag* and upstream *pol*, including a frameshift, were proposed to be used in expressing precursor polyproteins (*gag-pro* and *gag-pro-pol*) [23]. Nevertheless, amino acid sequences in this study demonstrated that *pro* domains were 100% identical between samples and Mn 92227. Comparison with baboon SERV, SRV1-5, and SRV-8 showed 83–88% identity (Table-3). Furthermore, *Pro* encodes part of a polyprotein (*gag-pro* or *gag-pro-pol*), whose products include a protease enzyme. As a result, it also functions to break down *gag* chains and other polyproteins to produce viral proteins in mature virions [20].

*Pol* is 2603 bp long and encodes 867 amino acids. The investigated amino acid sequences showed that the *pol* domain had 100% identity to the samples identified in this study and 99%–100% to Mn 92227. Comparison with baboon SERV, SRV1-5, and SRV-8, also showed an identity of 82%–88% (Table-3). Since *pol* is the coding domain part of a polyprotein (*gag-pro-pol*), its protein products are reverse transcriptase RT and integrase (IN) enzymes. The reverse transcriptase uses specific cellular tRNA primers to initiate negative-strand DNA synthesis and RNase-H-resistant PPT to initiate positive-strand DNA synthesis. Alternatively, integrase removes two bases from the end of an LTR and inserts a linear double-stranded DNA copy of a retroviral genome into the host cell DNA (proviral formation) [22].

However, *env* is 1776 bp long and encodes 591 amino acids. Although amino acid sequences showed that *env* domains were 100% identical between the obtained samples and Mn 92227, comparisons with baboon SERV and SRV1-5, including SRV8, exhibited 65%–79% identity (Table-3). *Env* is the binding target for antibodies produced by host immune cells for viral neutralization. Therefore, the virus makes changes in the *env* surface area to counteract the host immune system’s attack, accounting for the observed high variations in this area [18, 24, 25].

SERV U85505/1997 is an ERV from baboons (Papio), ungrouped with MnB1, MnB140910, and Mn 92227. Hence, we propose the possibility of a mutation in nucleotides that caused its amino acids to translate differently, resulting in a grouping shift in the phylogenetic tree. Studies have demonstrated the phylogeny tree for nucleotides and amino acids of Mn B1 and Mn B140910 samples always groups with Mn 92227. Similarly, our study showed that the samples obtained had 100% identity at the whole-genome sequence level (Table-3) with Mn 92227 NaPRC UW. This study indicates that Mn B1 and Mn B140910 had molecular characteristics, which we confirmed as novel SERV from pig-tailed macaque.

## Conclusion

Examination of blood samples from *M. fascicularis* and *M. nemestrina* at a primate breeding facility in Bogor, Indonesia, showed SERV in two samples from a pig-tailed monkey (Mn B1 and Mn B140910). Positive results were then characterized based on the SYBR Green real-time PCR’s TM values of 80°C–82°C and CT values of less than 24. Next, whole-genome sequencing revealed identical results with a total genome length of 7991 nucleotides for the two samples, which included LTR, *gag*, *pro*, *pol*, and *env* genes. These genes were subsequently used for genome mapping. Furthermore, the whole-genome sequence of Mn B1 and Mn B140910 had 99%–100% identity to an Mn 92227 isolate in NaPRC UW. Phylogenetic tree results also indicated that the samples at the whole-genome level clustered with Mn 92227 as a novel SERV. Based on these findings, we confirmed that SERV was detected in pig-tailed macaques from Indonesia. Therefore, it could be used in biomedical research on ERVs. Nevertheless, further studies should be conducted on known offspring animal samples to ensure that these SERVs are germline inherited.

## Authors’ Contributions

US, JP, and DI: Designed the study. FLNA and US: Conducted laboratory work and analyses. FLNA: Drafted and revised the manuscript. JP, US, and DI: Supervised the project. All the authors have read and approved the final manuscript.
